# *N*-(3-Chloro­phen­yl)benzene­sulfonamide

**DOI:** 10.1107/S1600536808026895

**Published:** 2008-08-23

**Authors:** B. Thimme Gowda, Sabine Foro, K. S. Babitha, Hartmut Fuess

**Affiliations:** a Department of Chemistry, Mangalore University, Mangalagangotri 574199, Mangalore, India; b Institute of Materials Science, Darmstadt University of Technology, Petersenstrasse 23, D-64287, Darmstadt, Germany

## Abstract

In the crystal structure of the title compound, C_12_H_10_ClNO_2_S, the N—H bond is *trans* to one of the S=O bonds. The two aromatic rings form a dihedral angle of 65.4 (1)°, compared with a value of 49.1 (1)° in *N*-(2-chloro­phen­yl)-benzene­sulfonamide. The mol­ecules are connected by inter­molecular N—H⋯O hydrogen bonds into chains running along the *b* axis.

## Related literature

For related literature, see: Gelbrich *et al.* (2007 ▶); Gowda *et al.* (2005 ▶, 2008*a* ▶,*b* ▶); Perlovich *et al.* (2006 ▶).
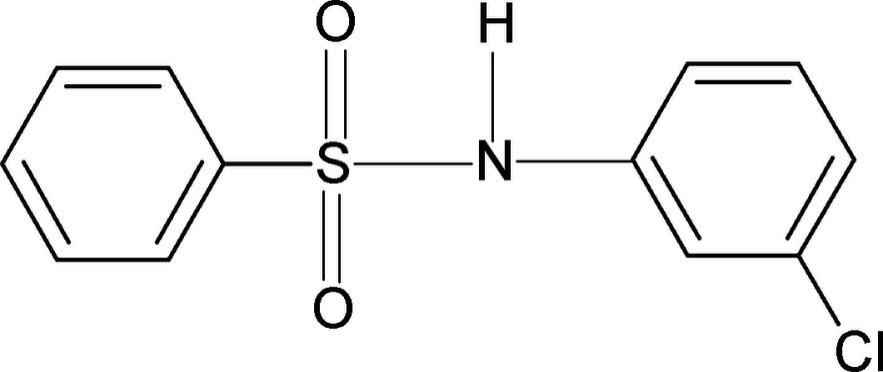


## Experimental

### 

#### Crystal data

C_12_H_10_ClNO_2_S
*M* *_r_* = 267.72
Tetragonal, 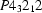

*a* = 8.8357 (7) Å
*c* = 32.081 (5) Å
*V* = 2504.6 (5) Å^3^
*Z* = 8
Cu *K*α radiation
μ = 4.18 mm^−1^
*T* = 299 (2) K
0.38 × 0.35 × 0.33 mm

#### Data collection

Enraf–Nonius CAD-4 diffractometer
Absorption correction: ψ scan (North *et al.*, 1968 ▶) *T*_min_ = 0.222, *T*_max_ = 0.251
5004 measured reflections
2232 independent reflections
2054 reflections with *I* > 2σ(*I*)
*R*_int_ = 0.071
3 standard reflections frequency: 120 min intensity decay: 1.0%

#### Refinement

*R*[*F* ^2^ > 2σ(*F* ^2^)] = 0.035
*wR*(*F* ^2^) = 0.094
*S* = 1.10
2232 reflections
158 parameters
19 restraints
H atoms treated by a mixture of independent and constrained refinement
Δρ_max_ = 0.19 e Å^−3^
Δρ_min_ = −0.21 e Å^−3^
Absolute structure: Flack (1983 ▶), 840 Friedel pairs
Flack parameter: −0.01 (2)

### 

Data collection: *CAD-4-PC* (Enraf–Nonius, 1996 ▶); cell refinement: *CAD-4-PC*; data reduction: *REDU4* (Stoe & Cie, 1987 ▶); program(s) used to solve structure: *SHELXS97* (Sheldrick, 2008 ▶); program(s) used to refine structure: *SHELXL97* (Sheldrick, 2008 ▶); molecular graphics: *PLATON* (Spek, 2003 ▶); software used to prepare material for publication: *SHELXL97*.

## Supplementary Material

Crystal structure: contains datablocks I, global. DOI: 10.1107/S1600536808026895/ci2659sup1.cif

Structure factors: contains datablocks I. DOI: 10.1107/S1600536808026895/ci2659Isup2.hkl

Additional supplementary materials:  crystallographic information; 3D view; checkCIF report

## Figures and Tables

**Table 1:** Hydrogen-bond geometry (Å, °)

*D*—H⋯*A*	*D*—H	H⋯*A*	*D*⋯*A*	*D*—H⋯*A*
N1—H1*N*⋯O2^i^	0.88 (1)	2.029 (13)	2.875 (2)	162 (2)

Symmetry code: (i) 
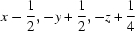
.

**Fig. 1.**
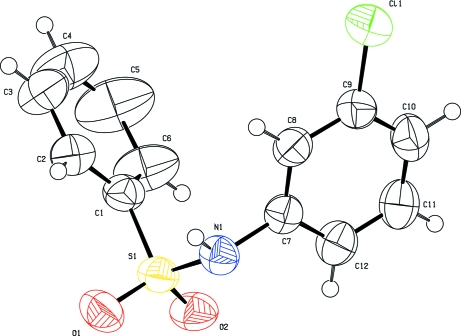
Molecular structure of the title compound, showing the atom labelling scheme. Displacement ellipsoids are drawn at the 50% probability level. H atoms are represented as small spheres of arbitrary radii.

**Fig. 2.**
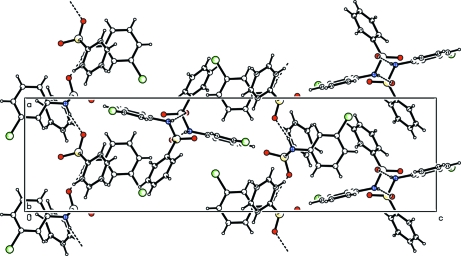
Molecular packing of the title compound, showing hydrogen-bonded (dashed lines) chains.

## supplementary crystallographic information

### Comment

As part of a study of the substituent effects on the crystal structures of *N*-(aryl)-benzenesulfonamides, in the present work, the structure of *N*-(3-chlorophenyl)-benzenesulfonamide (N3CPBSA) has been determined (Gowda *et al.*, 2008*a*,*b*). The N—H bond is trans to one of the S═O bonds (Fig. 1). Further, the conformation of the N—H bond is *anti* to the *meta*-chloro group in the aniline benzene ring, in contrast to the *syn* conformation observed with respect to the *ortho*-chloro group in *N*-(2-chlorophenyl)-benzenesulfonamide (N2CPBSA) (Perlovich *et al.*, 2006). The two benzene rings form a dihedral angle of 65.4 (1)° compared with the value of 49.1 (1)° in N2CPBSA. The other bond parameters in N3CPBSA are similar to those observed in N2CPBSA (Perlovich *et al.*, 2006) and other *N*-(aryl)-benzenesulfonamides (Gelbrich *et al.*, 2007; Gowda *et al.*, 2008*a*,*b*).

The packing diagram of N3CPBSA showing the N—H···O hydrogen bonds (Table 1) is shown in Fig. 2.

### Experimental

The solution of benzene (10 cc) in chloroform (40 cc) was treated dropwise with chlorosulfonic acid (25 cc) at 273 K. After the initial evolution of hydrogen chloride subsided, the reaction mixture was brought to room temperature and poured into crushed ice in a beaker. The chloroform layer was separated, washed with cold water and allowed to evaporate slowly. The residual benzenesulfonylchloride was treated with *m*-chloroaniline in the stoichiometric ratio and boiled for 10 min. The reaction mixture was then cooled to room temperature and added to ice cold water (100 cc). The resultant solid *N*-(3-chlorophenyl)-benzenesulfonamide was filtered under suction and washed thoroughly with cold water. It was then recrystallized to constant melting point from dilute ethanol. The purity of the compound was checked and characterized by recording its infrared and NMR spectra (Gowda *et al.*, 2005). Single crystals used in X-ray diffraction studies were grown in an ethanolic solution by evaporating it at room temperature.

### Refinement

The H atom of the NH group was located in a difference map and was refined with a N-H distance restraint of 0.90 (1) Å. The other H atoms were positioned with idealized geometry (C-H = 0.93 Å) and refined using a riding model with U_iso_(H) = 1.2*U*_eq_(C). The U^ij^ components of C4, C5 and C6 were restrained to approximate isotropic behaviour.

### Crystal data

**Table:**

C_12_H_10_ClNO_2_S	*Z* = 8
*M**_r_* = 267.72	*F*_000_ = 1104
Tetragonal, *P*4_3_2_1_2	*D*_x_ = 1.420 Mg m^−^^3^
Hall symbol: P 4nw 2abw	Cu *K*α radiation λ = 1.54180 Å
*a* = 8.8357 (7) Å	Cell parameters from 25 reflections
*b* = 8.8357 (7) Å	θ = 6.5–18.9º
*c* = 32.081 (5) Å	µ = 4.18 mm^−^^1^
α = 90º	*T* = 299 (2) K
β = 90º	Prism, colourless
γ = 90º	0.38 × 0.35 × 0.33 mm
*V* = 2504.6 (5) Å^3^	

### Data collection

**Table:**

Enraf–Nonius CAD-4 diffractometer	*R*_int_ = 0.072
Radiation source: fine-focus sealed tube	θ_max_ = 66.8º
Monochromator: graphite	θ_min_ = 5.2º
*T* = 299(2) K	*h* = −10→0
ω/2θ scans	*k* = −10→0
Absorption correction: ψ scan(North *et al.*, 1968)	*l* = −38→37
*T*_min_ = 0.222, *T*_max_ = 0.251	3 standard reflections
5004 measured reflections	every 120 min
2232 independent reflections	intensity decay: 1.0%
2054 reflections with *I* > 2σ(*I*)	

### Refinement

**Table:**

Refinement on *F*^2^	Hydrogen site location: inferred from neighbouring sites
Least-squares matrix: full	H atoms treated by a mixture of independent and constrained refinement
*R*[*F*^2^ > 2σ(*F*^2^)] = 0.035	*w* = 1/[σ^2^(*F*_o_^2^) + (0.0371*P*)^2^ + 0.1574*P*] where *P* = (*F*_o_^2^ + 2*F*_c_^2^)/3
*wR*(*F*^2^) = 0.094	(Δ/σ)_max_ = 0.004
*S* = 1.10	Δρ_max_ = 0.19 e Å^−^^3^
2232 reflections	Δρ_min_ = −0.21 e Å^−^^3^
158 parameters	Extinction correction: SHELXL97, Fc^*^=kFc[1+0.001xFc^2^λ^3^/sin(2θ)]^-1/4^
19 restraints	Extinction coefficient: 0.0031 (3)
Primary atom site location: structure-invariant direct methods	Absolute structure: Flack (1983), 840 Friedel pairs
Secondary atom site location: difference Fourier map	Flack parameter: −0.01 (2)

### Special details

**Table:**

Geometry. All e.s.d.'s (except the e.s.d. in the dihedral angle between two l.s. planes) are estimated using the full covariance matrix. The cell e.s.d.'s are taken into account individually in the estimation of e.s.d.'s in distances, angles and torsion angles; correlations between e.s.d.'s in cell parameters are only used when they are defined by crystal symmetry. An approximate (isotropic) treatment of cell e.s.d.'s is used for estimating e.s.d.'s involving l.s. planes.
Refinement. Refinement of *F*^2^ against ALL reflections. The weighted *R*-factor *wR* and goodness of fit *S* are based on *F*^2^, conventional *R*-factors *R* are based on *F*, with *F* set to zero for negative *F*^2^. The threshold expression of *F*^2^ > σ(*F*^2^) is used only for calculating *R*-factors(gt) *etc*. and is not relevant to the choice of reflections for refinement. *R*-factors based on *F*^2^ are statistically about twice as large as those based on *F*, and *R*- factors based on ALL data will be even larger.

### Fractional atomic coordinates and isotropic or equivalent isotropic displacement parameters (Å^2^)

**Table:**

	*x*	*y*	*z*	*U*_iso_*/*U*_eq_	
C1	−0.0696 (3)	−0.0170 (3)	0.09615 (8)	0.0566 (6)	
C2	−0.2118 (3)	−0.0569 (4)	0.10996 (9)	0.0763 (8)	
H2	−0.2603	0.0001	0.1304	0.092*	
C3	−0.2814 (4)	−0.1829 (4)	0.09306 (12)	0.1005 (11)	
H3	−0.3771	−0.2112	0.1023	0.121*	
C4	−0.2119 (5)	−0.2644 (4)	0.06353 (16)	0.1213 (15)	
H4	−0.2601	−0.3486	0.0523	0.146*	
C5	−0.0724 (5)	−0.2253 (5)	0.04988 (17)	0.1362 (17)	
H5	−0.0247	−0.2840	0.0297	0.163*	
C6	0.0006 (4)	−0.0978 (4)	0.06576 (12)	0.0987 (12)	
H6	0.0951	−0.0688	0.0558	0.118*	
C7	−0.0413 (3)	0.3249 (2)	0.05535 (7)	0.0518 (5)	
C8	−0.1736 (3)	0.3333 (3)	0.03316 (7)	0.0571 (5)	
H8	−0.2659	0.3123	0.0458	0.069*	
C9	−0.1672 (3)	0.3736 (3)	−0.00854 (8)	0.0633 (6)	
C10	−0.0304 (3)	0.4020 (3)	−0.02766 (8)	0.0685 (7)	
H10	−0.0269	0.4276	−0.0558	0.082*	
C11	0.1000 (3)	0.3923 (3)	−0.00488 (8)	0.0687 (7)	
H11	0.1925	0.4114	−0.0176	0.082*	
C12	0.0959 (3)	0.3547 (3)	0.03655 (8)	0.0633 (6)	
H12	0.1850	0.3494	0.0519	0.076*	
Cl1	−0.33446 (9)	0.38734 (11)	−0.03637 (3)	0.1003 (3)	
N1	−0.0492 (2)	0.2925 (2)	0.09923 (6)	0.0542 (5)	
H1N	−0.1414 (15)	0.300 (3)	0.1090 (7)	0.065*	
O1	−0.0183 (2)	0.1447 (2)	0.16226 (5)	0.0712 (5)	
O2	0.17669 (17)	0.1309 (2)	0.10738 (6)	0.0678 (5)	
S1	0.02122 (6)	0.13880 (7)	0.119048 (18)	0.05501 (19)	

### Atomic displacement parameters (Å^2^)

**Table:**

	*U*^11^	*U*^22^	*U*^33^	*U*^12^	*U*^13^	*U*^23^
C1	0.0552 (12)	0.0546 (11)	0.0599 (13)	0.0022 (9)	−0.0124 (11)	0.0011 (11)
C2	0.0731 (16)	0.0876 (19)	0.0681 (16)	−0.0161 (14)	−0.0063 (14)	0.0007 (15)
C3	0.092 (2)	0.093 (2)	0.117 (3)	−0.0326 (19)	−0.020 (2)	0.009 (2)
C4	0.109 (3)	0.075 (2)	0.181 (4)	−0.0087 (19)	−0.027 (3)	−0.033 (2)
C5	0.118 (3)	0.107 (3)	0.184 (4)	0.008 (2)	0.001 (3)	−0.087 (3)
C6	0.0725 (19)	0.098 (2)	0.126 (3)	0.0044 (15)	0.0010 (19)	−0.050 (2)
C7	0.0577 (12)	0.0460 (11)	0.0518 (12)	−0.0002 (9)	0.0055 (11)	−0.0063 (10)
C8	0.0565 (12)	0.0552 (12)	0.0595 (12)	−0.0069 (10)	0.0015 (11)	−0.0013 (11)
C9	0.0750 (15)	0.0592 (13)	0.0556 (13)	−0.0098 (12)	−0.0075 (12)	0.0017 (12)
C10	0.0926 (18)	0.0598 (13)	0.0532 (13)	−0.0039 (13)	0.0102 (14)	0.0022 (12)
C11	0.0671 (14)	0.0712 (15)	0.0679 (15)	0.0016 (12)	0.0192 (13)	0.0048 (13)
C12	0.0578 (12)	0.0658 (14)	0.0663 (14)	0.0007 (11)	0.0094 (12)	−0.0008 (13)
Cl1	0.0939 (5)	0.1250 (7)	0.0821 (5)	−0.0306 (5)	−0.0317 (4)	0.0267 (5)
N1	0.0536 (10)	0.0611 (10)	0.0481 (10)	0.0027 (8)	0.0041 (9)	−0.0041 (9)
O1	0.0762 (11)	0.0904 (12)	0.0470 (8)	0.0010 (10)	−0.0033 (8)	−0.0001 (9)
O2	0.0467 (8)	0.0850 (11)	0.0717 (11)	0.0016 (8)	−0.0091 (8)	−0.0047 (10)
S1	0.0492 (3)	0.0659 (3)	0.0499 (3)	−0.0007 (2)	−0.0058 (2)	−0.0026 (3)

### Geometric parameters (Å, °)

**Table:**

C1—C6	1.358 (4)	C7—N1	1.438 (3)
C1—C2	1.378 (4)	C8—C9	1.386 (3)
C1—S1	1.755 (2)	C8—H8	0.93
C2—C3	1.382 (4)	C9—C10	1.378 (4)
C2—H2	0.93	C9—Cl1	1.731 (3)
C3—C4	1.339 (5)	C10—C11	1.367 (4)
C3—H3	0.93	C10—H10	0.93
C4—C5	1.353 (6)	C11—C12	1.371 (4)
C4—H4	0.93	C11—H11	0.93
C5—C6	1.394 (5)	C12—H12	0.93
C5—H5	0.93	N1—S1	1.623 (2)
C6—H6	0.93	N1—H1N	0.876 (10)
C7—C8	1.371 (3)	O1—S1	1.4305 (17)
C7—C12	1.379 (3)	O2—S1	1.4256 (17)
			
C6—C1—C2	120.8 (3)	C9—C8—H8	120.7
C6—C1—S1	120.3 (2)	C10—C9—C8	120.9 (2)
C2—C1—S1	118.9 (2)	C10—C9—Cl1	120.41 (19)
C1—C2—C3	119.0 (3)	C8—C9—Cl1	118.73 (19)
C1—C2—H2	120.5	C11—C10—C9	119.3 (2)
C3—C2—H2	120.5	C11—C10—H10	120.3
C4—C3—C2	120.5 (3)	C9—C10—H10	120.3
C4—C3—H3	119.8	C10—C11—C12	120.8 (2)
C2—C3—H3	119.8	C10—C11—H11	119.6
C3—C4—C5	120.6 (4)	C12—C11—H11	119.6
C3—C4—H4	119.7	C11—C12—C7	119.6 (2)
C5—C4—H4	119.7	C11—C12—H12	120.2
C4—C5—C6	120.6 (4)	C7—C12—H12	120.2
C4—C5—H5	119.7	C7—N1—S1	122.09 (15)
C6—C5—H5	119.7	C7—N1—H1N	112.2 (16)
C1—C6—C5	118.4 (3)	S1—N1—H1N	106.4 (17)
C1—C6—H6	120.8	O2—S1—O1	119.45 (11)
C5—C6—H6	120.8	O2—S1—N1	107.89 (11)
C8—C7—C12	120.81 (19)	O1—S1—N1	104.81 (11)
C8—C7—N1	118.6 (2)	O2—S1—C1	107.00 (12)
C12—C7—N1	120.5 (2)	O1—S1—C1	108.81 (12)
C7—C8—C9	118.7 (2)	N1—S1—C1	108.50 (10)
C7—C8—H8	120.7		
			
C6—C1—C2—C3	1.4 (4)	C10—C11—C12—C7	0.7 (4)
S1—C1—C2—C3	−177.7 (2)	C8—C7—C12—C11	−0.4 (4)
C1—C2—C3—C4	−0.4 (5)	N1—C7—C12—C11	−176.9 (2)
C2—C3—C4—C5	0.3 (7)	C8—C7—N1—S1	115.6 (2)
C3—C4—C5—C6	−1.2 (8)	C12—C7—N1—S1	−67.8 (3)
C2—C1—C6—C5	−2.2 (5)	C7—N1—S1—O2	55.5 (2)
S1—C1—C6—C5	176.8 (3)	C7—N1—S1—O1	−176.22 (17)
C4—C5—C6—C1	2.1 (8)	C7—N1—S1—C1	−60.1 (2)
C12—C7—C8—C9	−0.5 (3)	C6—C1—S1—O2	−13.9 (3)
N1—C7—C8—C9	176.1 (2)	C2—C1—S1—O2	165.1 (2)
C7—C8—C9—C10	1.2 (3)	C6—C1—S1—O1	−144.3 (3)
C7—C8—C9—Cl1	−178.94 (17)	C2—C1—S1—O1	34.8 (2)
C8—C9—C10—C11	−1.0 (4)	C6—C1—S1—N1	102.2 (3)
Cl1—C9—C10—C11	179.2 (2)	C2—C1—S1—N1	−78.7 (2)
C9—C10—C11—C12	0.0 (4)		

### Hydrogen-bond geometry (Å, °)

**Table:**

*D*—H···*A*	*D*—H	H···*A*	*D*···*A*	*D*—H···*A*
N1—H1N···O2^i^	0.88 (1)	2.029 (13)	2.875 (2)	162 (2)

Symmetry codes: (i) *x*−1/2, −*y*+1/2, −*z*+1/4.
